# Facilitating a transparent and tailored scientific discussion about the added value of animal feeding trials as well as in vitro and in silico approaches with whole food/feed for the risk assessment of genetically modified plants

**DOI:** 10.1007/s00204-014-1375-7

**Published:** 2014-10-02

**Authors:** Joachim Schiemann, Pablo Steinberg, Bernard Salles

**Affiliations:** 1 Institute for Biosafety in Plant Biotechnology, Julius Kühn-Institut, Federal Research Centre for Cultivated Plants, Erwin-Baur-Str. 27, 06484 Quedlinburg, Germany; 2 Institute for Food Toxicology and Analytical Chemistry, University of Veterinary Medicine Hannover, Bischofsholer Damm 15, 30173 Hannover, Germany; 3 UMR 1331 INRA/INP/UPS, TOXALIM, 180 chemin de Tournefeuille, 31 027 Toulouse Cedex 3, France

The added value of animal feeding trials as well as in vitro and in silico approaches with whole food/feed for the risk assessment of genetically modified plants (GMPs) is a matter of persistent public debate when assessing the environmental and health risks. In the frame of two EU-funded projects (GRACE and G-TwYST) and several projects funded by European Member States (e.g., the French project GMO90+), animal feeding trials as well as in vitro and in silico approaches with whole GM food/feed are performed. To explore the added value of close cooperation and to reconsider the design, execution and interpretation of animal feeding trials as well as in vitro and in silico approaches, the above-mentioned projects agreed on exchanging material and data and are performing subchronic, chronic and carcinogenicity studies with two GM events in a highly coordinated manner. Other European research projects to investigate the added value of animal feeding trials for GMP risk assessment are invited to exchange material and data with us too.

We, the coordinators of the projects GRACE, G-TwYST and GMO90+ described below, agreed on a coordinated publication strategy to increase the transparency of our studies and—in addition to the stakeholder involvement activities in our own projects—to invite stakeholders to discuss the data generated by our projects in the frame of a scientific debate in an open-access journal. Therefore, we invite the Editor-in-chief of “Archives of Toxicology” to develop with us a process facilitating a transparent and tailored scientific discussion about the results of our research projects to be published. Our suggestion includes an open-access publication of the accepted GRACE, G-TwYST and GMO90+ manuscripts and further comprises the establishment of a discussion forum being maintained by the publisher. The forum should provide a platform for a solely evidence-based exchange of opinions, thereby objectifying the dispute about the adequacy of animal feeding trials with whole food/feed for the risk assessment of GMPs. The discussion forum will be opened with the publication of the first GRACE manuscript on two 90-day feeding trials, “90-day oral toxicity studies on two genetically modified maize MON810 varieties in Wistar Han RCC rats.”

GRACE (GMO Risk Assessment and Communication of Evidence) is a research project under the 7th Framework Program of the European Commission, starting in June 2012 with a duration of 3.5 years. It aims to increase the transparency, traceability and accessibility of information dealing with potential risks and benefits associated with the deliberate release of GMPs and products derived thereof. Besides the elaboration of systematic, transparent and inclusive reviews of existing evidence on potential health, environmental, and socioeconomic impacts of GMPs, one key objective is to reconsider the design, execution, and interpretation of animal feeding trials as well as in vitro and in silico approaches with whole food/feed and to determine their added value for the risk assessment of GMPs. Animal feeding trials with GM maize MON810 will cover a duration of 90 days and 1 year and will follow internationally accepted guidelines and recommendations. This central pillar of GRACE is of special importance in the light of the new Implementing Regulation (EU) No. 503/2013 on applications for EU market authorization of genetically modified food and feed. According to this Regulation, it is mandatory to perform a 90-day rodent feeding study for single transformation events, whereas “the Commission shall monitor the application of this Regulation, the developments in scientific knowledge on replacement, reduction and refinement of animal use in scientific procedures and the publication of new guidance from EFSA. The Commission shall in particular monitor the outcome of the research project called GRACE…”.^1^ Throughout the whole planning and interpretation stage, special emphasis is placed on active stakeholder involvement in order to consider the inputs from external experts on the scientific roadmap of GRACE, to increase transparency and to strengthen the relevance of the gathered results from a broader societal perspective (more detailed information on the GRACE stakeholder involvement strategy can be found on http://www.grace-fp7.eu/). Furthermore, all raw data will be made available for public scrutiny via the open-access database CADIMA (Central Access Database for the Impact Assessment of Crop Genetic Improvement Technologies, http://www.cadima.info/).

G-TwYST (GMP-Two Year Safety Testing) is also a research project under the 7th Framework Program of the European Commission, starting in April 2014 for a period of 4 years. G-TwYST will perform a 90-day as well as a combined 2-year chronic toxicity/carcinogenicity study with the GM maize NK603 and a 2-year carcinogenicity study with the GM maize MON810 based on OECD Test Guidelines and according to EFSA considerations. Furthermore, the project will define criteria to evaluate the scientific quality of long-term feeding studies and develop recommendations (scientific justification) on the added value of long-term feeding trials in the frame of the GMO risk assessment process. Partners will strictly comply with international standards and norms concerning feeding trials. Feeding stuff used in the trials will be produced according to the principles of good agricultural practice. Transparency and accessibility of project plans and results are a key characteristic of the project and will be ensured by establishing a project website and by using the open-access database (CADIMA) setup by GRACE as information hubs. The results will be published as open-access journal papers. By combining the results of the G-TwYST project with those of the GRACE project (90-day and 1-year study with the GM maize MON810), it will be possible for the first time to investigate potential medium-term and long-term toxic effects of the two above-mentioned events. G-TwYST will engage with stakeholders and inform the broader public and policy makers about the quality criteria for the evaluation and performance of feeding studies, their value and necessity, and in this context will discuss broader societal issues. The results of the project will enable risk managers drawing conclusions with regard to framework of the currently applicable GM food/feed risk assessment requirements and procedures in the EU.

GMO90+ (Genetically modified organism, 90-day to 180-day testing) is a research project aimed at improving the 90-day subchronic toxicity testing in rats. The project is supported by the Ministry of Ecology, Sustainable Development and Energy (MEDDE) with a grant of 2.5 million €, for a period of 3 years starting in 2014. GMO90+ will be unique as it brings together diverse scientific expertise in the area of crop production and analytical characterization, systems biology, conventional toxicology, statistics and modeling as well as social sciences and humanities. This project is based on a consortium of teams from laboratories at INRA (National Institute in Agronomy), INSERM (National Institute in Human Health), CNRS (National Institute in Scientific Research), ANSES (French Agency for Food Environmental and Occupational Health and Safety) and specialized partners in statistics, modeling, proteomics, and bioinformatics. The objective of this study is to investigate whether feeding rats with GM MON810 or NK603 maize may relate to biomarkers of effects. The GMO90+ consortium stresses the importance of longer studies conducted by GRACE and G-TwYST programs to confirm the predictive biomarkers identified in this project. The same crop production (fall 2014) as well as the same Wistar rat line will be used in the GMO90+ and G-TwYST/GRACE projects. Rats will be followed for 6 months (180 days) with monthly blood and urine samples allowing the search for biomarkers by high-throughput (omics) techniques in conjunction with pathophysiological analysis mainly centered on the gut, liver, kidney, and reproductive apparatus. The characterization and validation of biomarkers of interest will be discussed by the GMO90+ consortium in close relationship with the coordinators of the GRACE and G-TwYST programs. Results generated in the frame of GMO90+ should provide useful information to both academic research and regulatory agencies in order to improve the predictability of the 90-day rodent studies. The GMO90+ project is developed in a constant dialogue with stakeholders by insuring full transparency on this controversial research object. Finally, raw data will be loaded in the open-access database CADIMA, and frozen tissues will be stored and available in a first step for the GRACE and G-TwYST researchers and thereafter for the research community in general.

## Footnotes

1 Implementing Regulation (EU) (2013) Commission Implementing Regulation (EU) No. 503/2013 on applications for authorisation of genetically modified food and feed in accordance with Regulation (EC) No. 1829/2003 of the European Parliament and of the Council and amending Commission Regulations (EC) No. 641/2004 and (EC) No. 1981/2006. OJ L 157, 8.6.2013, pp. 1–48.

